# High‐risk older smokers' perceptions, attitudes, and beliefs about lung cancer screening

**DOI:** 10.1002/cam4.617

**Published:** 2016-01-28

**Authors:** Janine K. Cataldo

**Affiliations:** ^1^ University of CaliforniaSan FranciscoCalifornia; ^2^ UCSF Center for Tobacco ControlResearch and EducationSan FranciscoCalifornia

**Keywords:** Attitude to health, early detection of cancer/psychology, health status, lung neoplasms/diagnosis, older smokers, questionnaires, smoking/adverse effects

## Abstract

The US Preventive Services Task Force recommends that smokers aged 55–80 should be screened annually with low‐dose computed tomography (LDCT). This study identified demographics, smoking history, health risk perceptions, knowledge, and attitudes factors of older smokers (≥55 years) related to LDCT agreement. Using binary logistic regression, a predictive model of factors to explain LDCT agreement was produced. This is a cross‐sectional, national, online survey of 338 older smokers (≥55 years) with a ≥30 pack‐year smoking history. Over 82% of the sample believed that a person who continues to smoke after the age of 40 has at least a 25% chance of developing lung cancer and 77.3% would “agree to a LDCT today”. Using chi‐square analyses, six variables that were significant at the 0.10 level were selected for inclusion in model development. Four of the independent variables made a unique statistically significant contribution to the model: perceives accuracy of the LDCT as an important factor in the decision to have a LDCT scan; believes that early detection of LC will result in a good prognosis; believes that they are at high risk for lung cancer; and is not afraid of CT scans. Of note, only 10.9% believed that a negative CT scan result would mean that they could continue to smoke. Older smokers are aware of the risks of smoking, are interested in smoking cessation, and most are interested in and positive about LDCT. Cognitive aspects of participation in screening are key to increasing the uptake of lung cancer screening among high‐risk smokers.

## Introduction

Despite substantial reductions in smoking prevalence in the United States, lung cancer today is the leading cause of cancer death for both men and women 1. Of all lung cancer cases, more than 85% are caused by smoking and prognosis is poor, with mortality close to 90% 2. Among patients with cancer, patients with lung cancer have one of the lowest 5‐year survival rates (17%). For those whose cancer is diagnosed at an early stage, 5‐year survival is far better (52%), but currently, only 15% of all lung cancer cases are diagnosed at an early stage 3. Historically, these poor rates were due to the lack of effective routine screening. 4 This changed dramatically in November 2011 when the National Lung Cancer Screening Trial (NLST), the first large multicenter randomized control trial, released their results that showed that low‐dose computed tomography (LDCT) could reduce lung cancer mortality by 20% 5. LDCT is now widely recommended for current and former smokers. As of February 2015, The Centers for Medicare & Medicaid Services (CMS) determined that the evidence is sufficient to add a lung cancer screening counseling and shared decision‐making visit, and for appropriate beneficiaries, annual screening for lung cancer with LDCT, as an additional preventive service benefit under the Medicare program 6. The U.S. Preventive Services Task Force recommends that smokers and former smokers aged 55–80 who have a smoking history of 30 pack‐years or more, and who are currently smoking or have quit within the past 15 years, should be screened annually with LDCT 7.

Successful implementation of lung cancer screening depends on being able to reach high‐risk individuals. Studies show that those at higher risk are less interested in being screened despite recognizing that they are at risk 8, 9. Unlike screening programs for other cancers, this will be the first large‐scale screening program for a cancer that is so strongly associated with smoking. Silvestri (2011) found in a study of lung cancer screening attitudes that smokers, in comparison with nonsmokers, were less likely to want to be screened, had less understanding of the efficacy of screening, and found the cost prohibitive 8. Silvestri's study, however, predates the LCDT and subsequent guidelines for lung cancer screening. In a more recent study (2013), Tanner and colleagues found that smokers' fatalistic beliefs, fear of radiation exposure, and anxiety related to CT scans were associated with the decreased intention to agree to CT screening 10. However, this study was limited by its small sample size (*N* = 108) and they were from only one Veterans Administration Medical Center. There is a dearth of information on the attitudes and beliefs of smokers related to lung cancer screening 11. Identifying all barriers and facilitators for smokers is essential for promoting lung cancer screening for a high‐risk population.

In response to these important knowledge gaps, the purpose of this study was to survey a national sample of older smokers to describe perceptions and attitudes related to lung cancer screening. The aims for this study were to (1) describe older smokers' health risk beliefs related to cigarette smoking and lung cancer; (2) identify demographic, smoking history, health risk perceptions, knowledge, and attitude factors related to whether a smoker would agree to a LDCT scan; and (3) using binary logistic regression, provide a predictive model of factors to explain an older smoker's willingness to have a LDCT scan.

## Methods

As part of a larger Tobacco Attitudes and Beliefs Study, we conducted this cross‐sectional descriptive, correlational study with a national sample of older adult (age ≥55 years old) current and former smokers through the Qualtrics Research Company Panel. Qualtrics Panel is a probability‐based panel designed to be statistically representative of the U.S. population. Because all Qualtrics Panel households were selected randomly with a known probability of selection, Qualtrics Panel estimates can be used with the statistical confidence required.

Questions included demographic and clinical variables, past and present tobacco use experiences, and a panel of questions about attitudes and beliefs about lung cancer and lung cancer screening were developed by our research team and included in the survey (See Appendix A).

### Study population

In July 2014, a national Qualtrics Panel was conducted online. Eligible participants were current and former smokers over the age of 55. Based on the four CDC age divisions for smokers (i.e., Group 1, young adults 18–24; Group 2, adults 25–44; and older adults, Group 3 (45–64) and Group 4 (65 and older)) and the USPSTF recommendation based on 55 years old, for the purposes of this paper, we refer to older smokers as ≥55 years old. Current smoker was defined as at least 100 cigarettes in a lifetime and at least one cigarette in the last 7 days and former smoker was defined as at least 100 cigarettes in a lifetime but having successfully quit for less than 2 years. This web‐based survey was administered by Qualtrics, a commercial marketing research company, which aggregates online panels to create nationally representative samples from which to randomly select survey participants. A total of 11,676 individuals were invited to take the survey, of which 2186 clicked on the survey to yield a response rate of 18.7%. Participants were offered $10 for completing the survey. The final sample of 338 represented 46 out of 50 states, including a distribution across all four US Census Bureau Regions: 21.3% northeast, 23.9% midwest, 28.8% south, and 26.0% west. Before enrollment, the Committee on Human Research at the University of California, San Francisco, approved all planned study activities.

### Statistical analysis

Frequencies and descriptive statistics on all variables were generated. Chi‐square and *t*‐tests were used to identify differences between current and former smokers on all demographics and attitudes and beliefs variables. Pearson correlations among quantitative variables and chi‐square and Cramer's V for categorical variables were used to investigate associations among demographics, smoking factors, and attitudes and beliefs about LDCT. Univariate differences between groups for agreement and nonagreement for LDCT were conducted. Using all variables that demonstrated a significant association with LDCT agreement, a binary logistic regression analysis was conducted to predict agreement to have an LDCT.

## Results

The survey was completed online by 338 participants, the range of ages was 55 to 81, and mean age of the sample was 61.5 (SD 5.3). The mean years smoked was 41.2 (SD 9.4) and pack‐years ranged from 30 to 51. The sample included 314 (92.9%) current smokers, 175 (55.7%) of which were seriously considering cessation in the next 6 months and 65 (20.7%) in the next 30 days. The sample included 24 (7.1%) former smokers (quit <2 years); using chi‐square and *t*‐tests as appropriate, no differences were found between smokers and nonsmokers on any of the study variables.

As shown in Table 1, the sample was slightly more female (55.3%) and mostly Caucasian (87.3%). Over 82.2% of the samples were concerned about the long‐term health consequences of smoking cigarettes and 80.3% believed that smokers would live for “fewer years” than nonsmokers. When asked what percent chance a person who continues to smoke after the age of 40 would have of developing lung cancer, 82.0% of the sample believed that the person would have a 25–100% chance of developing lung cancer. None of the demographic variables were significantly associated with the decision to have a LDCT.

**Table 1:** Sample characteristics (*N* = 338)

	Range	M	SD
Age (≥55 years)	55–81	61.5	5.3
Years smoked	5–60	41.2	9.4
Mean pack‐years	30–52	31.1	4.6

	*n*	%
Current smoker^a^	314	92.9
Former smoker (quit <2 years)	24	7.1
Female	187	55.3
Caucasian/White	295	87.3
Income		
≤$10,000	27	38.0
$11–40,000	160	47.3
$41–70,000	92	27.2
$71–100,000	35	10.4
>$100,000	24	7.1
Education		
Did not complete high school	12	3.6
Completed high school	84	24.9
Some college	120	35.5
Completed college	86	25.4
Some graduate school	11	3.3
Completed graduate school	25	7.4
Employment		
Employed outside the home	131	38.8
Unemployed	31	9.2
Retired	150	44.4
Full‐time homemaker	23	6.8
Student	3	0.9

Data are presented as mean and standard deviation or %.

a Using *t*‐tests and chi‐square as appropriate, no significant differences were found between current and former smokers for all sample characteristics.

As shown in Table 2, 77.2% of the sample, if asked today, would agree to a LDCT. The majority 66.6% were “worried about LC” and 75.4% were “scared by thoughts of LC.” Only, 26.9% had been told by a clinician that they were at high risk of LC; yet, almost double that number (*n* = 176, 52.1%) believed that they were at high risk for LC. Overall, the beliefs about CT scans were positive: CT scan will decrease risk of dying from LC (67.2%), early detection of lung cancer will lead to a good prognosis (77.8%), and a negative result from a CT scan would decrease their worry of developing LC (53.6%). Of note, only 10.9% believed that a negative CT scan result would mean that they could continue to smoke. In their decision of whether to have a CT scan, 93.5% agreed that accuracy was important, followed by cost (78.4%), perception of risk of disease (77.2%), and convenience (74.9%).

**Table 2:** Perceptions, attitudes, and beliefs about LDCT (*N* = 338)

	Agree to LDCT today *n* = 261 (77.2%)	Not Agree to LDCT today *n* = 77 (22.8%)	χ^**2**^	*P*
	*n* (%)	*n* (%)		
Is worried about lung cancer	177 (67.8)	48 (62.3)	0.80	0.41
Is scared by thoughts of lung cancer	197 (75.5)	58 (75.3)	0.001	1.00
Believes CT radiation could cause lung cancer	107 (41.0)	25 (32.5)	1.82	0.19
Believes CT scan will decrease risk of dying from lung cancer	179 (68.6)	48 (62.3)	1.05	0.19
Believes a negative result CT will decrease worry of developing lung cancer	148 (56.7)	33 (42.9)	4.58	**0.04**
Believes a negative result CT does NOT mean they can continue to smoke without worrying	230 (88.1)	71 (92.2)	1.02	0.41
Believes CT scan is uncomfortable/painful	33 (12.6)	16 (20.8)	3.18	0**.10**
Is afraid CT scan will find cancer	134 (51.3)	44 (57.1)	0.80	0.44
Is NOT scared of CT scans	184 (70.5)	43 (55.8)	5.79	0.**02**
Is NOT nervous about CT scans	151 (57.9)	40 (51.9)	0.84	0.36
Has been told they are at high risk of lung cancer	75 (28.7)	16 (20.8)	1.91	0.19
Believes that they are at high risk for lung cancer	145 (55.6)	31 (40.3)	5.57	**0.02**
Believes that early detection of lung cancer will lead to a good prognosis	217 (83.1)	46 (59.7)	18.9	**<0.001**
Perception of importance of CT scanning convenience	199 (76.2)	54 (70.1)	1.18	0.30
Perception of importance of risk of disease	202 (77.4)	59 (76.6)	0.02	0.88
Perception of importance of screening accuracy	251 (96.2)	65 (84.4)	13.50	**0.001**
Perception of importance of screening cost	204 (78.2)	61 (79.2)	0.04	0.49

Significant at the 0.10 level were included in model development.

Using chi‐square analyses, six variables had a significant association with agreement to have a LDCT. Variables that were significant at the 0.10 level (Table 2) were selected for inclusion in model development. As shown in Table 3, only four of the independent variables made a unique statistically significant contribution to the model (perceives accuracy of the LDCT as an important factor in the decision to have a LDCT scan; believes that early detection of LC will result in a good prognosis; believes that they are at high risk for lung cancer; and is not afraid of CT scans). A test of the model against a constant only model was statistically significant, indicating that the predictors as a set, reliably distinguished between those who would agree to an LDCT and those who would not agree (χ^2^ = 41.00 *P *= <0.001 with df = 6). The model as a whole explained between 11.4% (Cox and Snell R square) and 17.4% (Nagelkerke R square) of the variance in agreement to have a CT scan, and correctly classified 80.5% of cases. As shown in Table 3, the strongest predictor was “perceives accuracy of CT scan as important factor in the decision to have a CT scan” (OR 3.0, 95% CI 1.13–7.95). Participants with this belief were three times more likely to agree to a CT scan than those who did not have the belief (controlling for all other factors in the model). The odds ratios for the remaining three predictors in the model were as follows: believes that early detection of LC will result in a good prognosis (OR 2.7, 95% CI 1.47–4.90); believes that they are at high risk for LC (OR 2.1, 95% CI 1.17–3.79); and is not afraid of CT scans (OR 0.41, 95% CI 0.23–0.75).

**Table 3:** Binary logistic regression LDCT agreement

Beliefs and perceptions	OR	95% CI for OR		*P*
		Lower	Upper	
Step 1				
Perceives accuracy of CT scan as important factor in the decision to have a CT scan	3.0	1.13	7.95	0.030
Believes that early detection of LC will result in a good prognosis	2.7	1.47	4.90	0.001
Believes that they are at high risk for LC	2.1	1.17	3.79	0.013
Is not afraid of CT scans	0.41	0.23	0.75	0.027
Constant	0.49			0.05

## Discussion

This study has several important findings: Older smokers are very concerned about the risks of lung cancer, almost 80% of the sample would agree to a LDCT. Four beliefs were strong predictors of agreement to LDCT: The LDCT scan is accurate; early detection of lung cancer will result in a good prognosis; they are at high risk for lung cancer; they are not afraid of CT scans; and having a CT scan will reduce their worry about developing lung cancer without encouraging continued smoking. Only about 11% believed that a negative CT scan result would mean that they could continue to smoke.

The findings in this study do not support previous studies that found that older smokers exhibit unrealistic optimism about their risks for tobacco‐related diseases 12, 13. Over 80% of the sample were concerned about the long‐term health consequences of smoking cigarettes and 77.5% believed that a person who continues to smoke after the age of 40 has at least a 25% chance of developing lung cancer (of note, 10% believed the person would have a 100% chance).

Current smoking has previously been identified as a barrier to LC screening 8, 10; however, in this study, almost 76.8% of older current smokers with at least 3 would agree to a LDCT. This is greater than a 2001 national telephone survey (71.7%) and less than a 2013 study of 209 veterans (100%). However, in the veteran study, sample size (*N* = 108), respondent bias was likely a problem, because the veterans surveyed were presenting for outpatient appointments, meaning that they had an established healthcare provider, in the current study, healthcare status is unknown. Not being able to identify a usual source of care is a known barrier to participation in screening programs 10.

In our study, 77.8% of the participants believed that early detection of LC would result in a good prognosis; those with this belief were almost three times more likely to agree to a CT scan than those without the belief). In a study of veterans who smoke by Tanner et al. 10, they evaluated “whether early detection of lung cancer results in a good chance of survival” and found that only 43.9% had this belief. In a second study, Jonnalagada et al 14. asked whether respondents agreed with the statement “CT scan will not decrease risk of dying,” they found no difference between groups with and without this belief on intention to screen. We asked two questions related to prognosis and risk, only 67.2% agreed that “CT scan will decrease risk of dying from LC.” However, 77.8% believed that “early detection of lung cancer would result in a good prognosis.” The difference in findings may be a result of difference in wording. In an attempt to put a more positive and less fatalistic frame to the question, we changed our question stem to “early detection of lung cancer will result in a good prognosis” therefore excluding the words “chance” and “survival.” This may have resulted in more affirmative answers.

Fear has been found to be a barrier to cancer screening 15, 16. In this study, there were three fear‐related items: scared of CT scans (32.8%), afraid radiation could cause lung cancer (39.1%), and afraid CT scan would find lung cancer (52.7%). However, only scared of CT scans was a significant independent predictor of whether a person would agree to a LDCT. This suggests that it is important to assess for general fear of screening CTs, not just fears related to lung cancer CT. Fears of CT scans have been promoted by the general media: one example is an article in *Consumer Reports* titled *“*The surprising dangers of CT scans and X‐rays*”* 17; in this article, radiation “overuse” is highlighted and readers are warned that “one scan leads to another.” Because of patient anxiety created by media inaccuracy and sensationalism, some patients will avoid imaging procedures 11.

In the present study, ethnicity was not associated with agreement to lung cancer screening; however, this may be a result of limited diversity (87.3% were Caucasian) and limited sample size (*N* = 338). Minority participants and Caucasian participants may hold different beliefs and attitudes toward screening. In previous studies, African Americans were more likely than whites to avoid an evaluation for lung cancer or be diagnosed later with more advanced lung cancer [18, 19, 20]. To ensure broad participation in lung cancer screening, knowledge of attitudes and beliefs about screening across racial and ethnic groups needs to be improved.

Previous studies found that cost of the LDCT was a barriers to willingness to be scanned 14 however, cost was not a significant concern in our study; whether the participants were covered by insurance was unknown. With the changing healthcare environment and the recent addition of Medicare coverage for LDCT, future studies need to assess healthcare coverage.

Almost 60% of the current smokers were considering cessation in the next 6 months and over 50% of all participants believed a negative CT scan would decrease their worry of developing lung cancer. Of note, only 10.9% believed that a negative LDCT would mean that they could continue to smoke without worry. Evidence is building that the provision of smoking cessation treatment in conjunction with annual lung cancer screenings has the potential to be a cost‐effective way to reduce tobacco use rates and smoking‐related morbidity and mortality in a vulnerable population, namely older smokers. There is support from professional organizations and insurers for LDCT sites to provide cessation treatment. As of 2010, an estimated 8.6 million Americans were eligible for LDCT screening 21. In lung cancer screening studies that included current and former smokers, approximately 50% of lung cancer screening participants were current smokers 22. LDCT lung cancer screening appears to provide an opportunity to deliver evidence‐based smoking cessation treatments to individuals who may be particularly receptive to a cessation intervention 22, 23. Lung cancer screening studies have found an increased motivation to quit smoking and higher rates of cessation among screening trial participants compared to spontaneous cessation in the general population, and a statistically significant increase in smoking cessation among individuals with an abnormal LDCT scan compared to those without abnormal results 22. Evidence‐based smoking cessation interventions in the LDCT setting can increase and leverage patients' motivation to quit smoking.

Limitations of this study are inherent to survey research and include the cross‐sectional nature of the study not allowing for causal inference and limited generalizability because of the difficulty accessing vulnerable populations. The results of this study are limited to a sample of older adult cigarette smokers aged ≥55 years that was not representative of the U.S. population. Study measures did not include whether the participants had insurance or Medicare, an important factor in lung cancer screening decisions. In addition, single items that assessed general attitudes and beliefs were not previously tested and therefore have limited validity. Strengths of this study included a moderately large (*N* = 338) national sample, and with forced response, missing data were not an issue. Although indicative of national online panel research, there was a relative low response rate of 18.7%.

In conclusion, older smokers are aware of the health risks of smoking, over half are considering cessation, they are interested in and open to lung cancer screening, and their overall beliefs about LDCT are positive. Cognitive aspects of participation in screening are key to increasing uptake of lung cancer screening among high‐risk smokers 24. Smokers need to not only be told they are at high risk for lung cancer, and they need to *believe* that they are at high risk for lung cancer and believe that early detection of lung cancer can result in an improved prognosis. Additional efforts should be taken to improve smokers' knowledge of the potential benefits and risks of LDCT when they are making decisions about participation in lung cancer screening 25. Effective smoking cessation interventions delivered in the LDCT setting could be a highly cost‐effective way to reduce smoking‐related morbidity and mortality.

## Conflicts of Interest

None.

## Supporting information


**Appendix S1.** Tobacco attitudes and beliefs study (with LDCT questions).
