# Supplementary material for: High‐risk older smokers' perceptions, attitudes, and beliefs about lung cancer screening
Source: Cancer Med. 2016 Jan 28;5(4):753–9. doi: 10.1002/cam4.617 (PMC4831294; doi:10.1002/cam4.617)
Supplement: Supplementary file 1 — Appendix S1. Tobacco attitudes and beliefs study (with LDCT questions). [file CAM4-5-753-s001.docx]

TOBACCO ATTITUDES AND BELIEFS STUDY (with LDCT questions)

Q2 Please answer the following screening questions.

|  | Answer | |
| --- | --- | --- |
|  | Yes (1) | No (2) |
| Are you 45 years old or older? (1) |  |  |
| Are you a current smoker? (2) |  |  |
| Are you a former smoker (quit smoking less than 2 years ago)? (3) |  |  |

If Are you 45 years old or old... Is Selected, Then Skip To End of BlockIf Are you a current smoker? -... Is Selected, Then Skip To End of BlockIf Are you a former smoker (qu... Is Selected, Then Skip To End of Block

Q3 TABS Tobacco Attitudes and Beliefs Study Thank you for participating in this important study about older adult heath and tobacco use. You will be asked questions about your life, about tobacco and related products, and about other health behaviors. These questions are to help us understand adult smokers better.   Your answers are extremely important to us, so please answer each question as honestly as you can. If you can’t remember or aren’t sure, just answer the best you can. There are no right or wrong answers.  Your survey answers will be handled as confidentially as possible. Your family and friends will not know how you answered the questions. Your name will not be used in any report or publication resulting from this study.   Please read all directions carefully. Many of the questions refer to a specific time period or situation or to certain tobacco products, and so it is important that you pay attention to the instructions, especially the words that are CAPITALIZED, IN BOLD PRINT, and ITALICS.   THANKS VERY MUCH.

Q4 Gender:

- Male (1)
- Female (2)
- Other (e.g., transgender) (3)

Q5 What ethnic group(s) best describes your background?

- African American / Black (1)
- Asian (2)
- Pacific Islander/Native Hawaiian (3)
- Caucasian / White (4)
- American Indian/Alaska Native (5)
- Hispanic or Latino (6)
- More than one ethnic group (7)
- Other (8)
- Not known (9)

Q6 Education (check highest level completed):

|  | Type of education (1) |
| --- | --- |
| No formal education (1) |  |
| Some grade school (2) |  |
| Completed grade school (3) |  |
| Some high school (4) |  |
| Completed high school / GED (5) |  |
| Some college (6) |  |
| Completed college (7) |  |
| Some graduate work (8) |  |
| Completed graduate degree (9) |  |

Q7 Number of years of completed education:

Q8 Occupational status:

- Employed (1)
- Unemployed (2)
- Retired (3)
- Full-time homemaker (4)
- Student (5)

Q9 Yearly individual income:

- Less than $10,000 (1)
- $11,000 – $20,000 (2)
- $21,000 – $30,000 (3)
- $31,000 – $40,000 (4)
- $41,000 – $50,000 (5)
- $51,000 – $60,000 (6)
- $61,000 – $70,000 (7)
- $71,000 – $80,000 (8)
- $81,000 – $90,000 (9)
- $91,000 – $100,000 (10)
- Over $100,000 (11)

Q10 What is your marital status

- Married (1)
- Live with intimate partner (2)
- Live with non intimate partner (3)
- Divorced (4)
- Separated (5)
- Widowed (6)
- Single, never married (7)

Q11 Does the person/s you live with smoke?

- No (1)
- Yes (2)
- I live alone (3)
- They are trying to quit (4)

Q12 In which state do you currently reside?

- Alabama (1)
- Alaska (2)
- Arizona (3)
- Arkansas (4)
- California (5)
- Colorado (6)
- Connecticut (7)
- Delaware (8)
- District of Columbia (9)
- Florida (10)
- Georgia (11)
- Hawaii (12)
- Idaho (13)
- Illinois (14)
- Indiana (15)
- Iowa (16)
- Kansas (17)
- Kentucky (18)
- Louisiana (19)
- Maine (20)
- Maryland (21)
- Massachusetts (22)
- Michigan (23)
- Minnesota (24)
- Mississippi (25)
- Missouri (26)
- Montana (27)
- Nebraska (28)
- Nevada (29)
- New Hampshire (30)
- New Jersey (31)
- New Mexico (32)
- New York (33)
- North Carolina (34)
- North Dakota (35)
- Ohio (36)
- Oklahoma (37)
- Oregon (38)
- Pennsylvania (39)
- Puerto Rico (40)
- Rhode Island (41)
- South Carolina (42)
- South Dakota (43)
- Tennessee (44)
- Texas (45)
- Utah (46)
- Vermont (47)
- Virginia (48)
- Washington (49)
- West Virginia (50)
- Wisconsin (51)
- Wyoming (52)
- I do not reside in the United States (53)

Q13 What type of community do you live in?

- Urban (1)
- Rural (2)
- Suburban (3)

Q14 Were you born in the United States?

- Yes (1)
- No (2)

Q15 What is your political party affiliation? Do you consider yourself a…

- Republican (1)
- Democrat (2)
- Independent (3)
- Other (4)

Q16 If you checked "Other" to the last question, please describe your political party affiliation here.

Q17  What brand of cigarettes do you smoke?

|  | Brand of |
| --- | --- |
|  | Cigarettes (1) |
| 1st (1) |  |
| 2nd (2) |  |
| 3rd (3) |  |

Q18 How many hours since your last cigarette?

Q19 How many cigarettes did you smoke in the last 24 hours?

Q20 How many cigarettes did you smoke in the 7 days prior to answering this questionnaire?

Q21 How many cigarettes a day do you smoke?

- 10 or less (1)
- 11-20 (2)
- 21-30 (3)
- 31 or more (4)

Q22 How soon after waking up do you have your first cigarette?

- After 60 minutes (1)
- 31-60 minutes (2)
- 6-30 minutes (3)
- Within 5 minutes (4)

Q23 Which cigarette would you most hate to give up?

- First cigarette in the morning (1)
- Cigarette during or after meals (2)
- Cigarette during or after stressful situations (3)
- Cigarette during social situations (4)
- None of the above (5)

Q24 Do you find it difficult to refrain from smoking in places where it is prohibited, such as in church, at the library, or in the movies?

- Yes (1)
- No (2)

Q25 Do you smoke more frequently during the first 2 hours of the day than during the rest of the day?

- Yes (1)
- No (2)

Q26 Do you smoke when you are so ill that you are in bed most of the day?

- Yes (1)
- No (2)

Q27 Has a health professional ever advised you to quit smoking?

|  | Answer |
| --- | --- |
|  | Y/N (1) |
| Yes (1) |  |
| No (2) |  |

Q28 How old were you when you first tried to smoke a cigarette?

Q29 How old were you when you started smoking cigarettes regularly?

Q30 How old are you now?

Q31 Date of Birth:  (MM/DD/YYYY)

Q32 How many years have you smoked?

Q33    What is the longest time you’ve ever stopped smoking?

Q34 In your lifetime, how many times have you quit smoking for 24 hrs or more?

Q35 In any previous quit attempts, which of the following methods / resources have you used to help you quit? (Check all that apply)

- Quit “cold turkey” (1)
- Gradually cut down (2)
- Stop smoking class/program for a fee (3)
- Stop smoking class/program (no fee) (4)
- Advice or counseling from MD, RN, psychologist or other health professionals (5)
- Telephone hotline (6)
- Hypnosis (7)
- Acupuncture (8)
- Nicotine gum (9)
- Nicotine patch (10)
- Nicotine spray (11)
- Nicotine inhaler (12)
- Nicotine lozenge (13)
- Zyban/Wellbutrin for smoking cessation (14)
- Chantix/Varenicline for smoking cessation (15)
- e-cigarettes (16)
- smokeless tobacco (17)
- Other (18)
- Never tried to quit (19)

Q36 If you selected "Other" for the last question, please describe/explain here:

Q37 In the last year, how many times have you quit smoking for 24 hrs or more? (Do not count hospital or incarceration)

Q38 Are you seriously considering quitting smoking within the next 6 months?

- Yes (1)
- No (2)

Q39 Are you planning to quit in the next 30 days?

- Yes (1)
- No (2)

Q162 In this section we want to know your thoughts about lung cancer and lung cancer screening. (Lung cancer screen = CT scan = CAT scan)

Q163 I am worried about lung cancer.

- True (1)
- False (2)

Q164 Thoughts of lung cancer scare me.

- True (1)
- False (2)

Q165 Do you consider the following statements about lung cancer and lung cancer screening TRUE or FALSE:

|  | Answer | |
| --- | --- | --- |
|  | True (1) | False (2) |
| Radiation from a CT scan could cause lung cancer (1) |  |  |
| CT scan will not decrease risk of dying from lung cancer (2) |  |  |
| CT scan with no lung cancer will decrease worry about developing cancer (3) |  |  |
| CT scan with no lung cancer means you can continue to smoke without worrying (4) |  |  |
| CT scan is uncomfortable/painful (5) |  |  |
| Afraid CT scan will find cancer (6) |  |  |
| CT scans scare me (7) |  |  |
| CT scans make me nervous (8) |  |  |
| You have been told by a doctor that you are at high risk for lung cancer (9) |  |  |
| You believe that you are at high risk for lung cancer (10) |  |  |
| If you were asked today – you would agree to a CT scan (11) |  |  |
| You believe that early detection of lung cancer will result in a good prognosis (12) |  |  |

Q166 In making decisions about whether to have a lung CT are the following important to you?  (questions 91-94):

|  | Answer | |
| --- | --- | --- |
|  | Important (1) | Not important (2) |
| Screening convenience (1) |  |  |
| Risk of disease (2) |  |  |
| Screening accuracy (3) |  |  |
| Screening cost (4) |  |  |

Q167 End of survey. Thank you so much for participating!
